# Towards understanding the initial performance improvement of PbS quantum dot solar cells upon short-term air exposure†

**DOI:** 10.1039/c8ra01422a

**Published:** 2018-04-20

**Authors:** Wenhui Gao, Guangmei Zhai, Caifeng Zhang, Zhimeng Shao, Lulu Zheng, Yong Zhang, Yongzhen Yang, Xuemin Li, Xuguang Liu, Bingshe Xu

**Affiliations:** Key Laboratory of Interface Science and Engineering in Advanced Materials of Ministry of Education, Research Centre of Advanced Materials Science and Technology, Taiyuan University of Technology Taiyuan Shanxi 030024 China zhaiguangmei@tyut.edu.cn; State Key Laboratory of Electronic Thin Films and Integrated Devices, University of Electronic Science and Technology of China Chengdu Sichuan 610054 China; Collaborative Innovation Centre for Advanced Thin-film Optoelectronic Materials and Devices in Shanxi Province Taiyuan Shanxi 030024 China; College of Materials Science and Engineering, Taiyuan University of Technology Taiyuan Shanxi 030024 China

## Abstract

An initial improvement in performance of PbS quantum dot solar cells composed of one thick layer of PbS quantum dots (QDs) treated with tetrabutylammonium iodide (PbS–TBAI) and one thin layer of PbS QDs capped with 1,2-ethanedithiol (PbS–EDT) over short-term air exposure is widely observed. However, the underlying mechanisms still remain elusive. In the work, we sought to understand the mechanisms as well as their physicochemical origins using a combination of X-ray photoelectron spectroscopy (XPS) and various electronic measurements. It is found that the PbS–TBAI film plays a dominant role in the initial device performance improvement compared with the PbS–EDT film. The PbS–TBAI film is compensation doped upon short-term air exposure (one to three days) owing to the increase of Pb–O and/or Pb–OH species, enabling its energy band to align better with the electron transport layer for more efficient charge extraction. Moreover, it is demonstrated that the short-term air exposure is capable of reducing defects in the devices and improving the diode quality, resulting in an initial increase in device performance. This work contributes to the fundamental understanding of the surface chemistry changes of PbS quantum dots treated by different ligands over air-exposure and the role of surface chemistry of quantum dots in optimizing their photovoltaic performance.

## Abstract 2

The mechanism at play that underpins the effect of short-term air exposure on the initial performance improvement of quantum dot solar cells is investigated.

## Graphical abstract

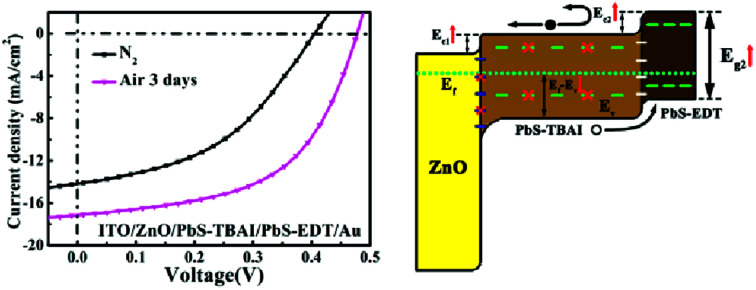

## Introduction

Colloidal quantum dots (QDs) are attracting increasing attention because their excellent properties including tunable bandgaps, broad-band light absorption, solution processability and potential multiple exciton generation effect make them suitable for low-cost next-generation electronic and optoelectronic device applications, especially photovoltaic devices.^1^ The efficiency of solution-processed solar cells based on lead chalcogenide colloidal QDs (*e.g.* PbS QDs) has increased from less than 1% to >10% over the past decade,^2^ dominantly enhanced *via* the subtle choice of QDs' surface ligands and the elaborate regulation of device configuration.^1,3^ To date a variety of short mono- and bidentate organic ligands including 1,2-ethanedithiol (EDT), mercaptocarboxylic acids (MPA) and 1,3-benzenedithiol (1,3-BDT) and monovalent inorganic atomic ligands such as tetrabutylammonium iodide (TBAI, I^−^) and tetrabutylammonium chloride (TBACl, Cl^−^) have been adopted to displace the original long alkyl ligands (*e.g.* oleic acid molecules) on the surface of QDs in order to boost the electronic coupling between neighboring QDs and deposit QD-films suitable for photovoltaic devices. Among the ligands, EDT and TBAI have become the most widely used organic and inorganic ligands for quantum dot solar cells (QDSCs), respectively, due to their short ligand length and/or strong affinity to cations on the QD surface. It has been reported that PbS QD-films passivated by EDT ligands are p-type semiconducting materials.^4,5^ However, PbS QD-films treated by TBAI usually exhibit an n-type doping character probably owing to two distinct mechanisms: (i) I^−^ anions substitute divalent S^2−^ anions within PbS QDs and (ii) I^−^ anions bound to PbS QD-surface repel oxidative attack.^6–9^ The tunability of colloidal QD solids' conduction (either n-type or p-type) offered by surface ligands realizes their full potential in the application of optoelectronic devices and makes it possible to fabricate high-performance QDSCs by changing device architectures to the greatest extent.

So far quantum dot solar cells with a variety of device structures including Schottky QDSCs,^10,11^ heterojunction QDSCs,^12–14^ quantum junction QDSCs^6,15^ and so on have been fabricated to improve device performance. Recently, Bawendi *et al.*^16^ reported that not only device performance but also stability in quantum dot solar cells can be enhanced by engineering the band alignment of two quantum dot layers through the use of TBAI and EDT ligand treatments in combination. Therein, a much thicker layer of iodide-passivated PbS QDs (hereinafter referred to as PbS–TBAI film) serves as the main light-absorbing layer and a thin layer of EDT-passivated PbS QDs (hereinafter referred to as PbS–EDT film) serves as an electron-blocking/hole-extraction layer. This device configuration has been widely adopted to fabricate state-of-the-art PbS QDSCs at present. Differing from PbS QDSCs with single EDT ligands,^17^ the devices with TBAI and EDT bi-ligands exhibit much better long-term storage stability in air. Nevertheless, same as single-ligand devices,^17,18^ these devices also generally show an initial increase in power conversion efficiency after short-term air exposure,^16^ which has been observed in some other labs including ours as well.^19^ The increase in device performance is always desirable. However, the underlying mechanism still remains elusive.

We take the view that understanding and identification of mechanisms at play in the initial device performance improvement is essential for further device optimization. Thus, we sought to understand the mechanisms at work that underpin the effects of short-term air exposure on device performance as well as their physicochemical origins using a combination of X-ray photoelectron spectroscopy (XPS) and various electronic measurements in the work. It is found that the PbS–TBAI film plays a dominant role in the initial device performance improvement compared with the PbS–EDT film. The PbS–TBAI film is compensation doped upon short-term air exposure (1–3 days' air exposure) owing to the increase of Pb–O and/or Pb–OH species, enabling its energy band to align better with the electron transport layer for more efficient charge extraction. Moreover, it is demonstrated that the short-term air exposure is capable of reducing surface defects in the devices and improving the diode quality, resulting in the initial increase in device performance. This work contributes to the fundamental understanding of the surface chemistry changes of PbS quantum dots treated by different ligands over air-exposure and the role of surface chemistry of quantum dots in optimizing their photovoltaic performance.

## Experimental

### Materials

PbO (99.9%), oleic acid (90%), 1-octadecene (ODE, 90%), and 1,2-ethanethiol (98+%) were purchased from Alfa Aesar. Hexamethyldisilathiane (synthesis grade), tetrabutylammonium iodide (≥98.0%) and acetonitrile (99.8%) were ordered from Sigma-Aldrich. Methanol (≥99.9%) was purchased from Aladdin. Zn(CH_3_COO)_2_·2H_2_O (≥99%), KOH, *n*-butanol, chloroform, and anhydrous acetone were obtained from Sinopharm Chemical Reagent Co., Ltd. Poly (3,4-ethylenedioxythiophene) poly (styrenesulfonate) (PEDOT : PSS) was purchased from Xi'an Polymer Light Technology Corp. LiF (99.99%), Al (99.999%), Ag (99.99%) and Au (99.999%) were ordered from Zhongnuo Advanced Material Technology Co., Ltd. All these chemicals were used as received unless stated otherwise.

### Synthesis of colloidal PbS QDs

Colloidal PbS QDs with an excitonic absorption peak in octane at ∼950 nm (as shown in Fig. S1 in the ESI†) were synthesized following a previously reported method with minor changes.^18,20^ Specifically, a mixture of lead oxide (2 mmol, 446 mg), oleic acid (5 mmol, 1.6 ml) and 1-octadecene (20 ml) in a three-neck flask was heated and degassed under vacuum at 95 °C for at least 6 hours. And then, the clear lead-oleate solution was heated up to 120 °C using a heating mantle under N_2_ atmosphere. 210 μl of hexamethyldisilathiane (1 mmol) was carefully mixed with 10 ml of ODE in a dry N_2_-filled glove-box. We note that the ODE had been degassed under vacuum at 90 °C for 2 hours before mixing with hexamethyldisilathiane. Then the ODE solution of hexamethyldisilathiane was rapidly injected into the three-neck flask and the solution turned black immediately. After 20 seconds, the dark solution was moved from the heating mantle and then cooled to room temperature. All of the above procedures were carried out under magnetic stirring until the reaction was completed. The as-synthesized PbS QDs were isolated by adding anhydrous acetone and then centrifuging, purified by dispersion/precipitation with toluene/methanol three times, and finally dispersed in octane with a concentration of 40 mg ml^−1^ for solar cell fabrication.

### Synthesis of ZnO nanoparticles

The synthesis of ZnO nanoparticles is similar to the method reported previously.^21^ Briefly, Zn(CH_3_COO)_2_·2H_2_O (2.95 g, 13.4 mmol) was dissolved in 125 ml of methanol with stirring at 60 °C. A solution of KOH (1.48 g, 23 mmol) in methanol (65 ml) was then slowly added into the zinc acetate solution and the solution was kept stirring at 65 °C for 2.5 hours. After cooling to room temperature, the supernatant was decanted and the precipitate washed twice with methanol (20 ml). Finally, n-butanol (70 ml), methanol (5 ml) and chloroform (5 ml) were added to disperse the precipitate and produce a ZnO-nanoparticle solution.

### Device fabrication

All devices including solar cells, Schottky devices (for capacitance–voltage measurements) and single carrier devices mentioned in the work were fabricated on indium-tin-oxide (ITO) coated glass substrates (sheet resistance: 13–17 Ω □^−1^). The ITO substrates were cleaned sequentially by ultrasonication in detergent, de-ionized water, acetone, and ethanol for 15 min, respectively. The substrates were blown dry with nitrogen and exposed to ultraviolet (UV)/ozone radiation for 10 min before use. Layer-by-layer spin-coating was applied to fabricate PbS QD films in air. The synthesized PbS QDs in octane were firstly spin-casted at 2500 rpm for 15 s onto some substrate to form a QD layer. Then the layer was exposed to either TBAI (10 mg ml^−1^ in methanol) or EDT (0.02% v/v in acetonitrile) for 30 s before spin drying and finally rinsed twice with the solvent of the ligand and spin-dried. The process was repeated until the desired thickness was achieved. For solar cells, the PbS–QD layer was deposited on top of a low-temperature processed ZnO-nanoparticle film (90 nm). The ZnO-nanoparticle film was deposited by spin-coating ZnO-nanoparticle solution onto the ITO substrate at 2500 rpm for 30 s and then annealed on a hot plate at 130 °C for 10 min in air. Finally, an Au electrode (0.5–1 Å s^−1^) was deposited on top of the PbS–QD film by thermal evaporation to complete device fabrication. The effective area of cells was defined as 3 mm^2^. For Schottky devices, the active layer was deposited on top of a PEDOT : PSS film (50 nm) which was formed by spin-casting PEDOT : PSS solution in water and annealing at 150 °C for 15 min. The fabrication of Schottky devices was finished by thermal evaporation of 1 nm LiF (0.1 Å s^1^) and 80 nm Al (1–3 Å s^−1^) at a base pressure of around 3 × 10^−6^ torr. For single carrier devices, 80 nm of Ag (0.5–1 Å s^−1^) was deposited on top of the PbS–QD film as electrode contact by thermal evaporation under vacuum of ∼10^−6^ torr. It should be noted that all films were deposited in air and stored overnight in dry air (≤15% RH) unless stated otherwise before being moved into a N_2_-filled glovebox where electrode layers were deposited *via* thermal evaporation.

### Characterizations

The current density–voltage (*J*–*V*) characteristics of solar cells and single carrier devices were measured using a Keithley 2400 source-meter in N_2_ atmosphere at room temperature. The AM 1.5G illumination of 100 mW cm^−2^ was calibrated by using a standard Si reference cell certificated by the National Institute of Metrology, China. In air exposure experiments, the solar cells and single carrier devices were first tested in a N_2_-filled glovebox, then the devices were moved out of the glovebox and exposed to air in a dry cabinet (≤15% RH) for one to three days before further measurements in the glovebox. The morphology of the PbS quantum dots was measured using a JEOL JEM-2010 transmission electron microscope (TEM). The optical absorbance spectra of the PbS QD films were recorded by a Perkin Elmer Lambda 950 UV-Vis-NIR spectrometer. The changes in surface chemistry of the PbS QD films with the same air exposure process as devices were characterized by X-ray photoelectron spectroscopy (XPS, Amicus, Kratos). The PbS QD films for optical absorbance and XPS tests were prepared on glass substrates in air unless stated otherwise. In order to mimic the device air exposure process, these films were also stored in vacuum of ∼10^−6^ torr for one hour before optical absorbance and XPS measurements. The XPS spectra were calibrated to the C 1s peak at a binding energy of 284.8 eV. Capacitance–voltage (*C*–*V*) curves of the Schottky devices were characterized by a Keithley 4200 system equipped with a Lakeshore probe station in the dark. *C*–*V* sweeps were performed between −3 and 5 V and the AC signal was set to 30 mV and 5 KHz. Given the sensitivity of LiF to water vapour in the air, the *C*–*V* measurement of some Schottky devices including LiF was performed in a dry oxygen atmosphere to accelerate the oxidization of the PbS–QD films.

## Results and discussion

**Fig. 1:**
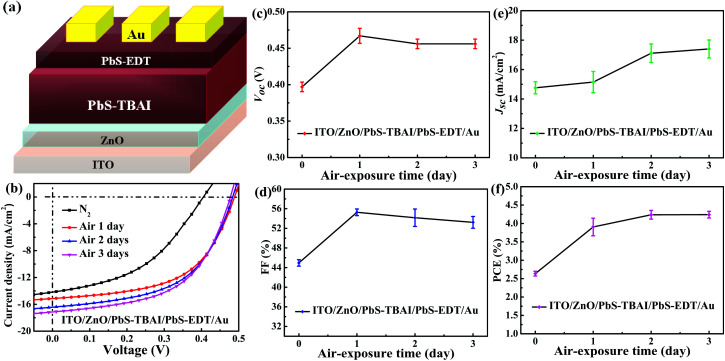
(a) The device architecture of PbS quantum dot solar cells fabricated in the work. (b) Typical light *J*–*V* characteristics of the devices with different air-exposure time under simulated AM1.5G irradiation (100 mW cm^−2^). (c–f) The evolution of performance parameters of the devices with air-exposure: (c) open circuit voltage (*V*_OC_), (d) fill factor (FF), (e) short-circuit current density (*J*_SC_), (f) power conversion efficiency (PCE). The average (symbols) and standard deviation (error bars) were calculated from a sample of six devices on the same substrate.

Fig. 1 shows the evolution of photovoltaic parameters with air exposure time in devices treated with TBAI and EDT ligands (PbS–TBAI/PbS–EDT devices) schematically shown in Fig. 1(a), as well as their typical *J*–*V* curves in light. It can be clearly seen from Fig. 1(b)–(f) that all four device parameters including open circuit voltage (*V*_oc_), short circuit current density (*J*_sc_), fill factor (FF) and power conversion efficiency (PCE) exhibit an increase after one day of air exposure, especially obvious for *V*_oc_, FF and PCE, which is similar to the previous report.^16^ As the air exposure time is prolonged to three days, PCE gradually increases from initial 2.46% to 4.25% (average values) in accompany with the enhanced *J*_sc_, while *V*_oc_ and FF remain essentially unchanged. We note that afterwards the devices can stay relatively stable with no obvious performance changes for more than one month before degradation (Fig. S2†). For the devices with single TBAI or EDT ligands (namely the PbS–TBAI devices and PbS–EDT devices), as shown in Fig. S3(a) and (b) in the ESI,† they display distinct evolutionary trends of device performance. Thereinto, all parameters of the PbS–TBAI devices gradually increase with air exposure time, while the PbS–EDT devices quickly degrade after air exposure, which is the same as our previous results.^17^ Actually, as pointed out previously,^18^ the PbS–TBAI devices usually need longer air-exposure time to reach their highest efficiency. Obviously, the PbS–TBAI/PbS–EDT devices behave very similarly to the PbS–TBAI devices rather than the PbS–EDT devices, implying that the PbS–TBAI film might play a dominant role in their performance change in comparison with the PbS–EDT layer due to the former's much higher thickness (200 nm as opposed to 40 nm for the PbS–EDT layer).

**Fig. 2:**
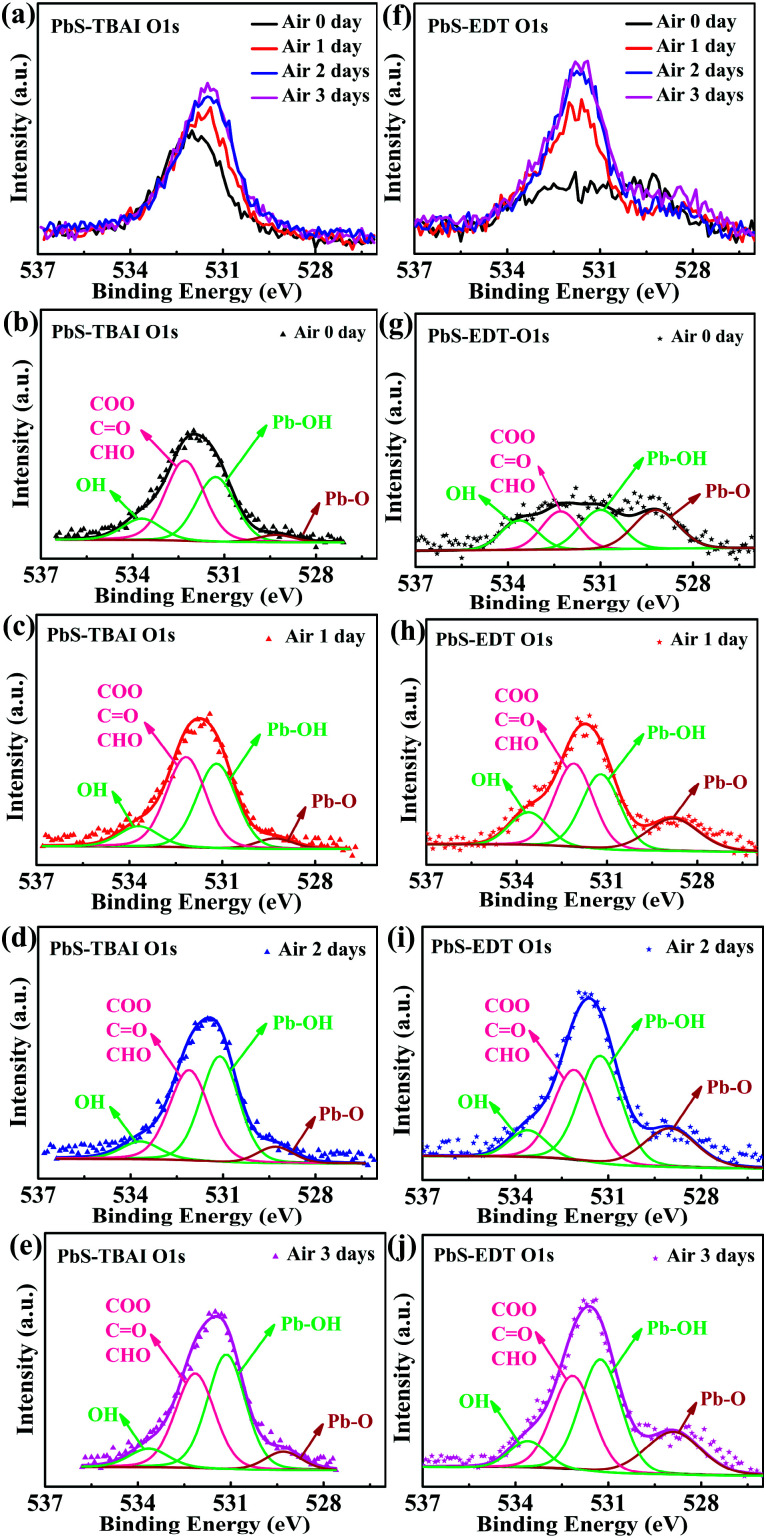
(a) XPS spectra of the O 1s core level of the PbS–TBAI film with different air-exposure time. Deconvoluted XPS spectra of (b) the PbS–TBAI film before air exposure, (c) the PbS–TBAI film after one day of air exposure, (d) the PbS–TBAI film after two days of air exposure, (e) the PbS–TBAI film after three days of air exposure; (f) XPS spectra of the O 1s core level of the PbS–EDT film with different air-exposure time. Deconvoluted XPS spectra of (g) the PbS–EDT film before air exposure, (h) the PbS–EDT film after one day of air exposure, (i) the PbS–EDT film after two days of air exposure, (j) the PbS–EDT film after three days of air exposure. Note: XPS spectra of the O 1s core level of the PbS–TBAI and PbS–EDT films cannot be compared with each other directly.

**Fig. 3:**
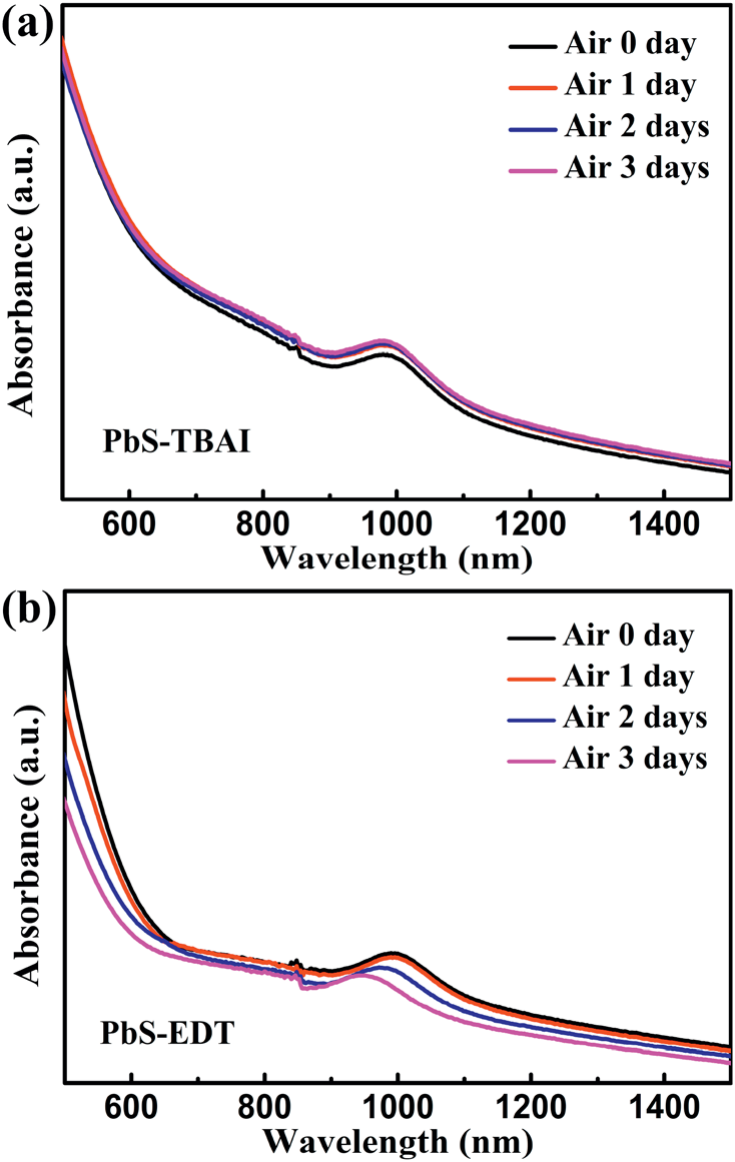
Evolution of absorption spectra of (a) the PbS–TBAI film and (b) the PbS–EDT film with air exposure time. The discontinuity at wavelength of 850 nm is an artifact resulting from detector changeover.

To identify the origin of the improved photovoltaic performance after short-term air exposure, we firstly explored the surface changes of the PbS–TBAI and PbS–EDT films with air-exposure time *via* XPS. The O 1s core level spectra of the PbS–TBAI and PbS–EDT films at different air-exposure time are shown in Fig. 2. The spectra can be deconvoluted into four peaks: the peak at 531.2 eV can be assigned to the hydroxyl ligand binding to Pb on the surface of PbS QDs, namely Pb–OH species;^9,22,23^ the peak at 532.2 eV is associated with COO, C=O and/or CHO groups in residual (partially oxidized) oleate ligands; ^24,25^ the side peak at 529.3 eV is attributed to Pb–O species;^26^ the side peak at 533.7 eV may be traceable to OH of residual methanol molecules in the PbS–TBAI film or OH resulting from deprotonation of SH moieties through a reaction with hydroxides for the PbS–EDT film.^25^ The details of the deconvolution parameters are tabulated in Table S1 in the ESI.† It can be seen from Fig. 2(b) and (g) that some Pb–OH species and Pb–O species had existed in the PbS–TBAI and PbS–EDT films before air exposure because they were fabricated and stored in air overnight (the same procedure as that for device fabrication) before the XPS measurement. Fig. 2(a) and (f) clearly show that further oxidization and hydroxylation of the PbS QDs indeed happened for both films after air exposure, while the effect of air exposure on the PbS–EDT film is much more aggressive than that on the PbS–TBAI film, which is in agreement with their absorption evolution with air-exposure time, as shown in Fig. 3. For the PbS–TBAI film, as shown in Fig. 2(b)–(e), the intensities of the peaks associated with Pb–OH and Pb–O species increased obviously after two days' air exposure, suggesting that the surface of PbS–TBAI QDs has been significantly hydroxylated and oxidized in the first two days. Nevertheless, no further apparent changes can be observed afterwards. As shown in Fig. 3(a), the absorption peak of the PbS–TBAI film shows no obvious shift, indicating the effective size (*i.e.* the energy bandgap, *E*_g_) of the PbS QDs capped with I^−^ anions does not change. Therefore, we believe that the occurred Pb–OH and Pb–O species do not form an insulating layer on the surface of PbS-QDs capped with I^−^ anions due to the strong Pb–I interaction. Otherwise, the size of the QDs would reduce and result in an apparent blue-shift of the absorption peak for the PbS–TBAI film. These Pb–OH and Pb–O species newly formed in the PbS–TBAI film with air exposure may more likely serve as p-type dopants and defect passivation agents as reported previously.^25,27^ For the PbS–EDT film, its surface chemistry shows a more drastic change after one day of air exposure as compared with the PbS–TBAI film. Except for the significant hydroxylation and oxidization with increasing air exposure time similar to the PbS–TBAI film, a sharp intensity increase of the peak at 532.2 eV related to residual oleate ligands after one day of air-exposure can be also observed in the PbS–EDT film, which is distinct from the PbS–TBAI film. It is probably due to the fact that acetonitrile is used as ligand solvent and rinsing solvent for the EDT ligand-exchange rather than methanol in the case of the TBAI exchange. It has been reported that methanol can strip oleate ligands on QDs while acetonitrile cannot,^28,29^ causing more residual oleate ligands to be oxidized in the PbS–EDT film than in the PbS–TBAI film. In addition, the increased intensity of the peak assigned to unbounded thiols (SH) as well as concurrently decreased intensity of the peak attributed to bound thiolates with air exposure time (Fig. S4 and Table S2 in the ESI†)^25,30^ suggests EDT ligands can be desorbed from the surface of PbS QDs probably due to weak Pb–thiol binding. As shown in Fig. 3(b), the absorption spectra of the PbS–EDT film shows a monotonic decrease in intensity and an obvious blue shift of the excitonic absorption peak with air exposure time, which in combination with the XPS results indicates an insulating layer composed of Pb–OH and Pb–O species should be formed on the surface of the PbS QDs capped with EDT due to weak Pb–thiol binding, thereby decreasing the effective size of these PbS QDs.^17,26^ However, the intensity of the O 1s core level spectra of the PbS–EDT film is no longer significantly increased after two days of air exposure, which is inconsistent with its gradual decrease in absorption with air exposure time. We speculate it could be due to the small sampling depth of our XPS instrument which has been exceeded by the thickness of insulating layers formed on the PbS–EDT QDs.

**Fig. 4:**
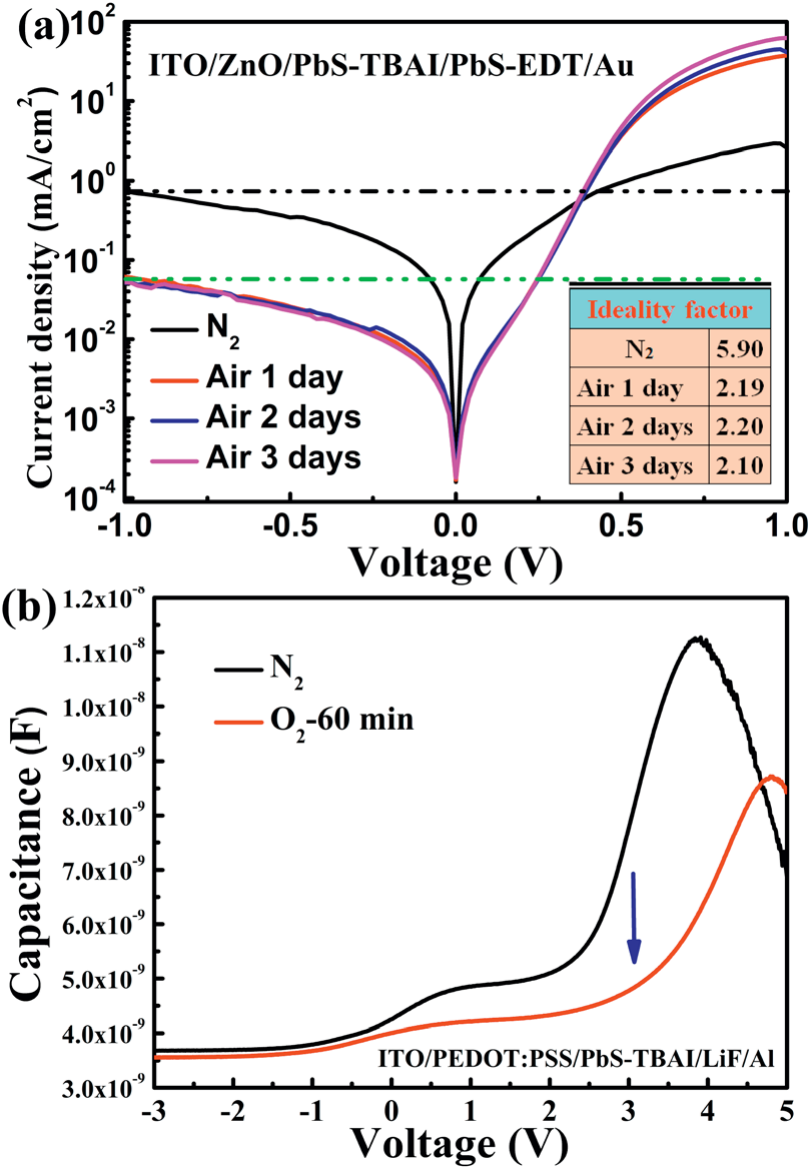
(a) Dark *J*–*V* characteristics of the PbS quantum dot solar cells with different air-exposure time. Inset: ideality factors extracted from the dark *J*–*V* curves; (b) capacitance–voltage curves of the device with a structure of ITO/PEDOT : PSS/PbS–TBAI/LiF/Al before air exposure and after one hour of oxygen exposure.

To obtain more information on the PbS–TBAI/PbS–EDT devices, we further analyzed their dark *J*–*V* curves, as shown in Fig. 4(a). The extracted diode ideality factors (*n*) from the dark *J*–*V* curves at a forward applied voltage of 0.21–0.36 V are plotted in the inset of Fig. 4(a). The value of *n* was found to be dramatically decreased from 5.9 before air exposure to 2.19 after one day of air exposure and then remains almost unchanged over the following two days of air exposure, implying that the diode quality can be improved by appropriate air-exposure. High ideality factors (much larger than 2) have been observed in other diodes and have been attributed to parasitic rectifying junctions within the device.^31^ Considering there are two main junctions (a ZnO/PbS–TBAI heterojunction and a PbS–TBAI/PbS–EDT homojunction) within a PbS–TBAI/PbS–EDT device, we believe that the high ideality factors obtained here for the devices before and after air exposure are reasonable. Nevertheless, the ideality factor is also associated with recombination mechanisms. It is generally thought that ideality factors greater than 2 can be attributed to a complex recombination process with traps distributed in the devices.^32^ In our case, the higher value of *n* (∼5.9) for the device before air-exposure indicates it may suffer severe trap-assisted recombination, whereas the dramatically decreased ideality factor (∼2.19) after one day of air exposure, we believe, may be related to the alleviation of recombination as a consequence of the passivation of quantum dots with the increase of Pb–O and Pb–OH species upon air exposure (as evidenced by the XPS analysis above). The almost unchanged values of *n* after longer air exposure indicate that the traps/defects cannot be further reduced. Moreover, as can be seen from Fig. 4(a), the reverse saturation dark current density for the PbS–TBAI/PbS–EDT device without air-exposure is much higher than that of the devices after air exposure. The higher reverse saturation dark current is also caused by the higher concentration of traps/defects, which is correlated with the limitation of *V*_oc_ and FF. The significantly decreased reverse saturation dark current of more than one order of magnitude after air exposure may account for the increased *V*_oc_ according to the equation^33^*V*_oc_ = *nk*_B_*T*/*q* ln(*J*_sc_/*J*_0_), where *J*_0_ is the reverse saturation dark current density, *q* the elementary charge, *k*_B_ the Boltzmann constant, and *T* the temperature. In order to further confirm the reduction of traps/defects after short-term air exposure, we used a capacitance based spectroscopic technique,^34^*i.e.* capacitance–voltage (*C*–*V*) measurement, to explore defects in the main optical absorbing layer (the PbS–TBAI film) with the diode structure of ITO/PEDOT : PSS/PbS–TBAI/LiF/Al. In forward bias, in addition to depletion capacitance and diffusion capacitance, there is another contribution to capacitance due to the presence of trapped charges, namely trap capacitance. The traps can capture electrons as forward bias increases, resulting in increased capacitance, but the capacitance will drop eventually due to finite number of traps in the active layer, leading to the occurrence of peak capacitance at some voltage larger than the built-in potential.^34^ As shown in Fig. 4(b), the device's capacitance is greatly decreased after one hour of oxygen-exposure (accelerated oxidization), further corroborating the reduction of traps/defects in the PbS–TBAI film after short-term air exposure. The passivation effect on the PbS QD surface that eliminates trap centres due to some degree of oxidation has also been observed in other groups.^10,35,36^ However, more traps/defects seem to be introduced into the PbS–EDT layer on the air-exposure time scale we studied here, as shown in Fig. S5,† further indicating that the device performance is mainly determined by the properties of the PbS–TBAI layer.

**Fig. 5:**
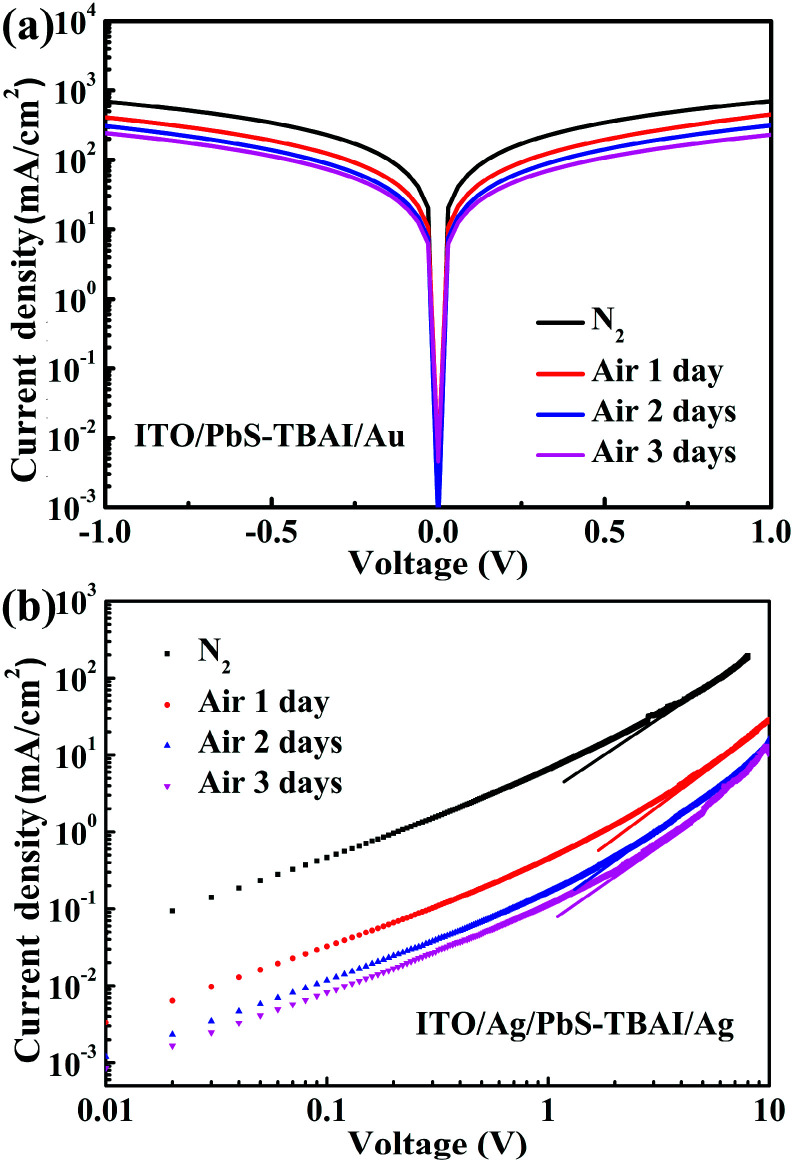
(a) Dark *J*–*V* curves of the device with a structure of ITO/PbS–TBAI/Au after different air-exposure time; (b) space charge limited current measurements of the device with a structure of ITO/Ag/PbS–TBAI/Ag after different air-exposure time.

To reveal the effect of air exposure on electronic properties of the PbS–TBAI film, its conductivity was further studied by measuring dark *J*–*V* curves of a simple device with the structure of ITO/PbS–TBAI/Au. The evolution of dark *J*–*V* characteristics of the PbS–TBAI film with air exposure time is shown in Fig. 5(a). It is clear that the conductivity of the PbS–TBAI film gradually decreases from 1.43 × 10^−3^ S m^−1^ to 4.66 × 10^−4^ S m^−1^ though the decrease becomes smaller and smaller with air exposure time. Considering that PbS–QD solids treated by TBAI usually exhibit n-type conduction,^6,8,9,16^ we believe that the decreased conductivity of the PbS–TBAI film after air exposure is understandable because its n-type conduction may be partly compensated by compensation doping of the newly formed p-type dopants including the Pb–OH and Pb–O species. We also extracted electron mobilities of the PbS–TBAI film with different air-exposure time by measuring the space charge limited current (SCLC) of a single-electron device with the structure of ITO/Ag/PbS–TBAI/Ag according to the method reported previously.^4,37^ The results are shown in Fig. 5(b). The decreased electron mobility from initial 9.8 × 10^−3^ cm^2^ V^−1^ s^−1^ to 4.4 × 10^−4^ cm^2^ V^−1^ s^−1^ with air exposure further corroborates the compensation doping of the PbS–TBAI film. Additionally, the compensation doping should result in a downward shift of the Fermi level (*E*_f_) with respect to the valence band edge (*E*_v_) of the PbS–TBAI film. The consequence of this downward shift of the Fermi level is an increase in the energy difference between the Fermi levels of the separate ZnO and PbS–TBAI films, leading to a larger build-in potential at the ZnO/PbS–TBAI heterojunction in the PbS–TBAI/PbS–EDT device. The increased build-in potential can significantly extend the width of the depletion region in the PbS–TBAI layer taking into account the much higher ZnO doping density than the PbS doping density,^16,38^ resulting in the improvement of the charge-extraction efficiency and thereby the increased short-circuit current. The enhanced fill factor after air exposure is a further benefit of the increased depletion region.

**Fig. 6:**
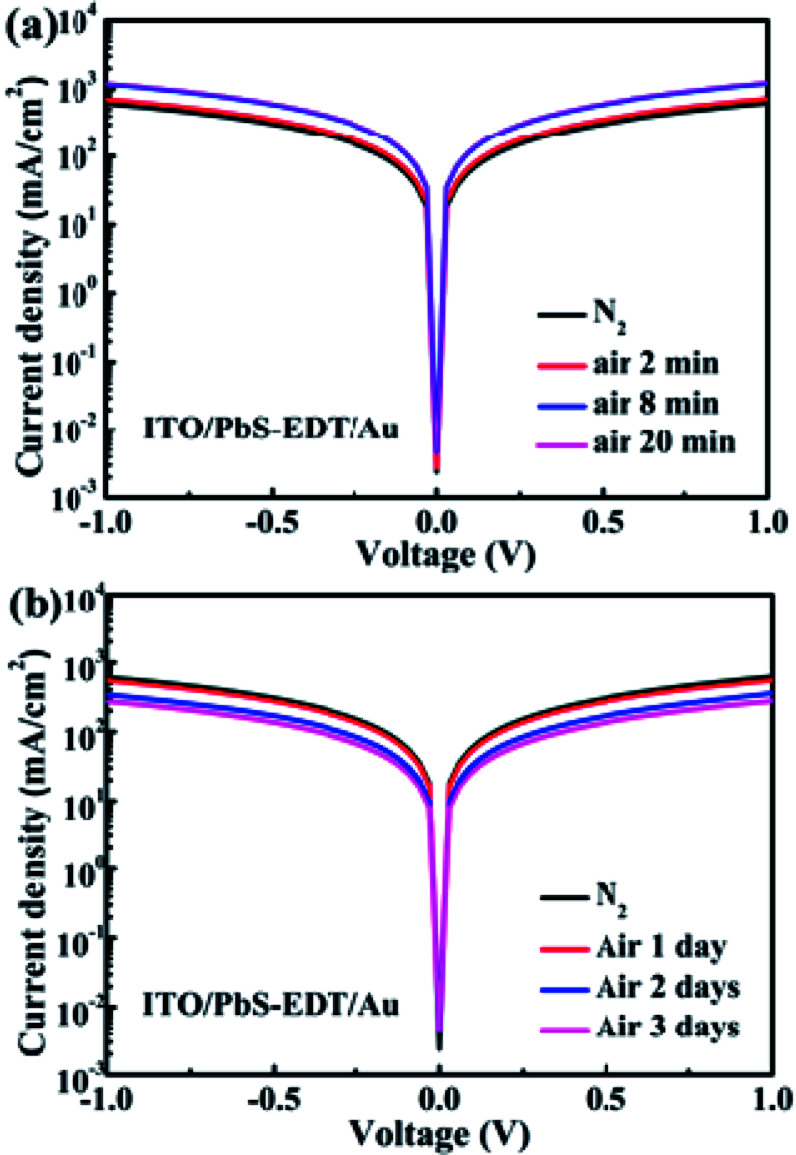
(a) Dark *J*–*V* curves of the device with a structure of ITO/PbS–EDT/Au after two to twenty minutes of air exposure (note: the blue curve and the pink curve are overlapped); (b) dark *J*–*V* curves of the device with a structure of ITO/PbS–EDT/Au after one to three days of air exposure.

As demonstrated previously,^16^ in the PbS–TBAI/PbS–EDT device the PbS–EDT layer serves as an electron-blocking/hole-extraction layer between the PbS–TBAI layer and the gold contact through proper band alignment, leading to the improved photocurrent and enhanced device performance in comparison with the PbS–TBAI device. The interfacial band bending at the PbS–TBAI/PbS–EDT homojunction only makes an additional minor contribution to the improved *J*_sc_. In order to further explore the change at the PbS-TABI/PbS–EDT homojunction with air exposure, the conductivity of the PbS–EDT film with different air-exposure time was also measured. We can see from Fig. 6(a) that the conductivity of the PbS–EDT film was improved after only several minutes of air-exposure. It can be ascribed to the effective p-type doping and/or defect passivation as a result of the formation of Pb–OH and Pb–O species on the surface of the PbS–EDT QDs. However, when the air-exposure time was further prolonged to one to three days (the time scale we are concerned about in this work), its conductivity gradually decreased. Combining with the XPS and optical absorbance results shown above, we believed that the decreased conductivity is due to the formation of insulting layers on the surface of the PbS–EDT QDs, which can impede carrier transport in the film. Therefore, we think that the improved device performance after one to three days of air exposure for the PbS–TBAI/PbS–EDT devices cannot be mainly ascribed to the PbS–EDT film, although the shorter-term (several minutes) air exposure may be beneficial to the device enhancement due to appropriate p-type doping and/or defect passivation as reported previously.^17^ On the contrary, we believe that the PbS–EDT layer is the Achilles' heel of the PbS–TBAI/PbS–EDT devices with respect to their long-term device stability, and the severe hydroxylation and oxidization of this layer could cause the final device degradation. Of course, owing to the small thickness of the PbS–EDT layer as well as the existence of a depletion region in the PbS–EDT layer, the charge transport in this layer will not be severely influenced during the early stage of the device lifecycle. Moreover, the increased energy band gap (*E*_g_, mainly upward shifted conduction band) due to oxidization could make a marginal contribution to the improved *J*_sc_.

**Fig. 7:**
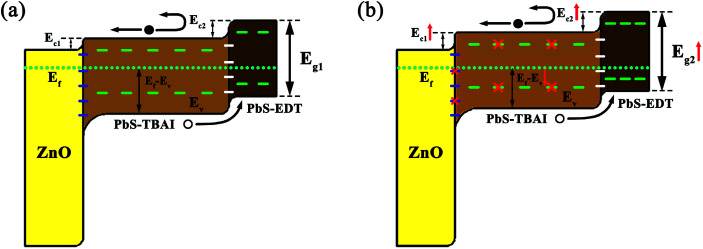
Schematic illustration of suggested energy band diagram of the PbS–TBAI/PbS–EDT device before (a) and after (b) short-term air exposure. Note: Δ*E*_c_ shown here just stands for the difference between conduction band positions of these semiconductors before contact with each other. The “dashed lines” in the schematic are to guide the eye and indicate trap states, however their relative locations do not represent actual positions of these trap states.

Based on these observation and analysis we suggest a model for the mechanism through which the device performance was initially improved, as shown in Fig. 7. Before air exposure, the PbS QDs are characterized by a number of trap states on their surface throughout their bandgap; the Fermi level of the PbS–TBAI QDs is more closer to the conduction band (n-type conduction) while the PbS–EDT QDs' Fermi level is more closer to the valence band (p-type conduction). The resulting schematic energy band diagram of the PbS–TBAI/PbS–EDT device in equilibrium is shown in Fig. 7(a). After one to three days of air exposure, the newly formed Pb–OH and Pb–O species passivate part of the trap states in the PbS–TBAI layer and compensation dope the n-type PbS–TBAI QDs, leading to diminished trap states, improved built-in potential, extended depletion region, larger conduction band offsets (Δ*E*_c_, simply determined by two separate conduction band positions) and thereby the enhanced device performance including *V*_oc_, *J*_sc_, FF and PCE, as schematically shown in Fig. 7(b).

## Conclusions

In the work, we have demonstrated the possible mechanisms at play that underpin the improved performance of PbS quantum dot solar cells incorporating one PbS–TBAI layer and one PbS–EDT layer after short-term air exposure (one to three days), as well as their physicochemical origins using a combination of X-ray photoelectron spectroscopy and various electronic measurements. It is found that the PbS–TBAI film plays a dominant role in the initial device performance improvement. The PbS–TBAI film is compensation doped upon short-term air exposure owing to the increase of Pb–O and/or Pb–OH species, enabling their energy bands to align better with the electron transport layer for more efficient charge extraction. Moreover, it is demonstrated that the short-term air exposure is capable of reducing defects in the devices and improving the diode quality, resulting in the initial increase in device performance. This work suggests that the quantum dot solar cells with higher performance and longer lifetime may be expected through better individual doping and passivation control on these two quantum-dot layers by ligands and moderate air-exposure in combination. We do note, however, that further work is needed to fully understand the mechanism which is driving the initially enhanced solar cell performance.

## Conflicts of interest

There are no conflicts of interest to declare.

## Supplementary Material

RA-008-C8RA01422A-s001
