# Supplementary material for: Towards understanding the initial performance improvement of PbS quantum dot solar cells upon short-term air exposure
Source: RSC Adv. 2018 Apr 20;8(27):15149–57. doi: 10.1039/c8ra01422a (PMC9080000; doi:10.1039/c8ra01422a)
Supplement: RA-008-C8RA01422A-s001 [file RA-008-C8RA01422A-s001.pdf]

Electronic Supplementary Material (ESI) for RSC Advances.
This journal is © The Royal Society of Chemistry 2018

Supporting Information

Towards understanding the initial performance improvement of PbS quantum dot solar cells upon short-term air exposure

Wenhui Gao,[a,c] Guangmei Zhai,*[a,b,c] Caifeng Zhang,[a,c] Zhimeng Shao,[a,c] Lulu Zheng,[a,c] Yong Zhang,[a,c,d] Yongzhen Yang,[a,c] Xuemin Li,[a,c] Xuguang Liu,[a,c,d] and Bingshe Xu[a,c]

[a.] *Key Laboratory of Interface Science and Engineering in Advanced Materials of Ministry of Education, Research Centre of Advanced Materials Science and Technology, Taiyuan University of Technology, Taiyuan, Shanxi 030024, China. E-mail: zhaiguangmei@tyut.edu.cn*

[b.] *State Key Laboratory of Electronic Thin Films and Integrated Devices, University of Electronic Science and Technology of China, Chengdu, Sichuan 610054, China*

[c.] *Collaborative Innovation Centre for Advanced Thin-film Optoelectronic Materials and Devices in Shanxi Province, Taiyuan, Shanxi 030024, China*

[d.] *College of Materials Science and Engineering, Taiyuan University of Technology, Taiyuan, Shanxi 030024, China*

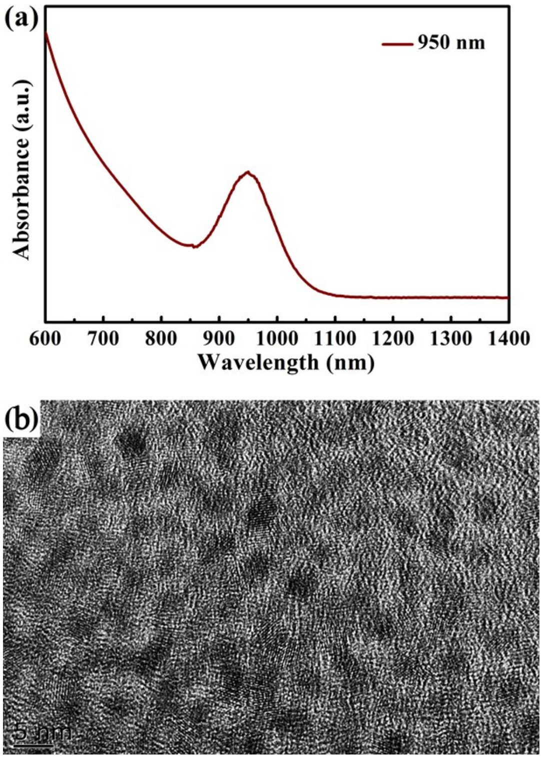

Fig. S1 (a) The absorption spectrum of the PbS quantum dot solution in octane used in the work. (b) A TEM image of the PbS quantum dots synthesized in the work.

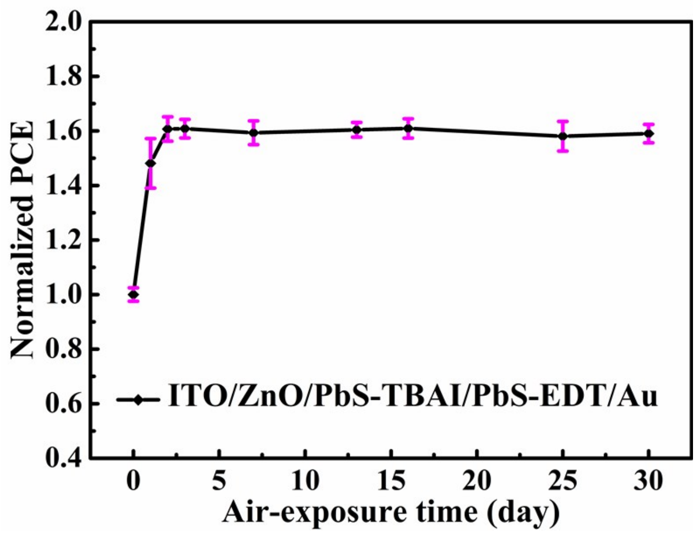

Fig. S2 The typical performance evolution of PbS quantum dot solar cells with TBAI and EDT bi-ligands upon air-exposure.

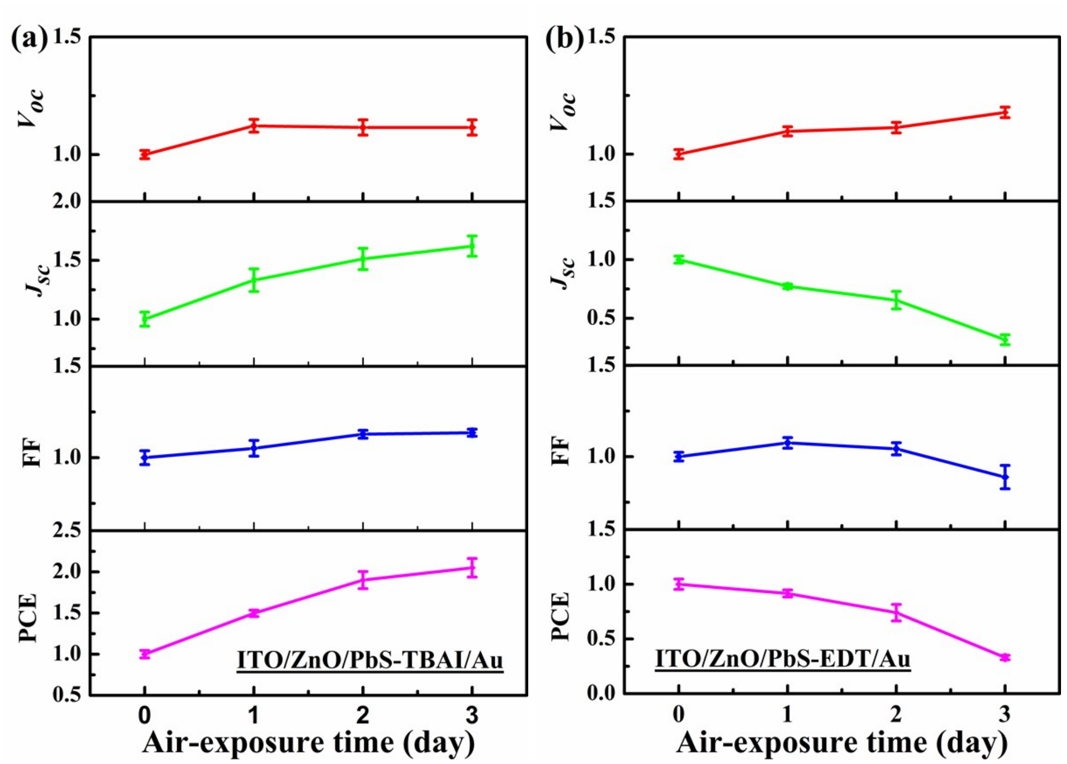

Fig. S3 The evolution of normalized performance parameters of (a) the devices based on PbS QD-films treated by TBAI only, (b) the devices based on PbS QD-films treated by EDT only.

Table S1 The parameters used for deconvolution of the O1s core level spectra of the PbS-TBAI and PbS-EDT films.

The PbS-TBAI film

component	peak (eV)	FWHM (eV)
Pb-O	529.3	1.4
Pb-OH	531.2	1.5
COO,C=O,CHO	532.2	1.5
OH	533.7	1.5

The PbS-EDT film

component	peak (eV)	FWHM (eV)
Pb-O	529.3	2
Pb-OH	531.2	1.5
COO,C=O,CHO	532.2	1.5
OH	533.7	1.5

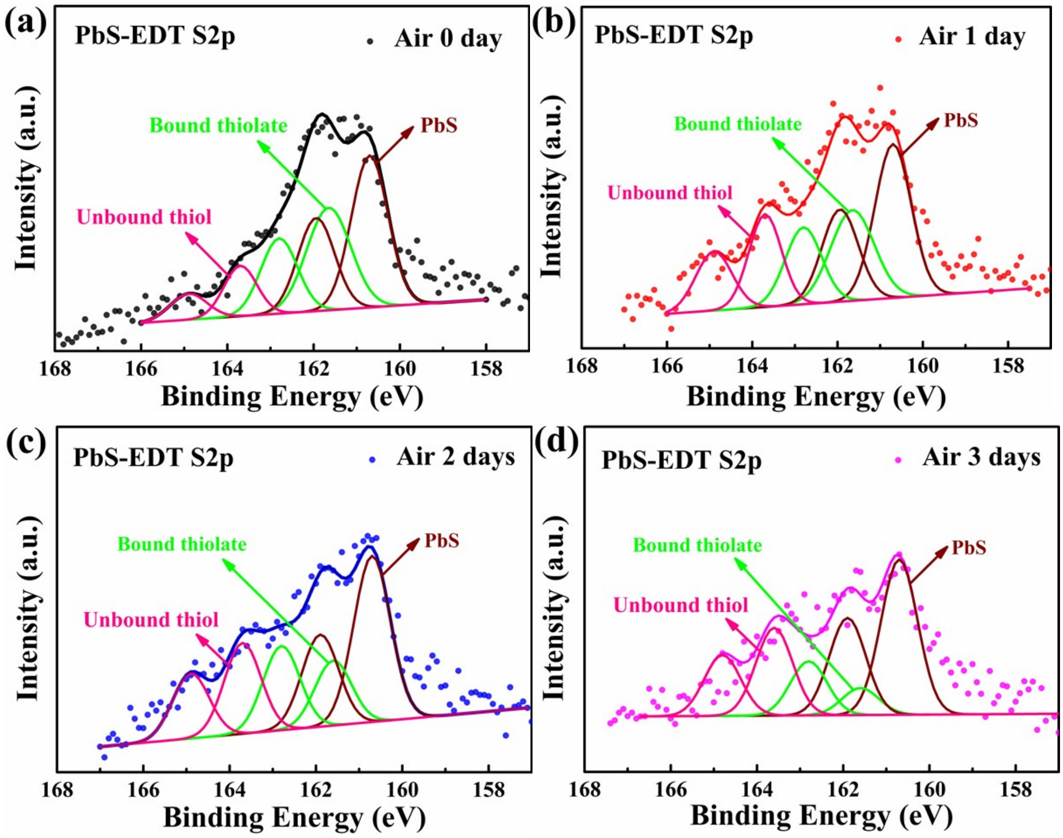

Fig. S4 Deconvoluted XPS spectra of the S2p core level of the PbS-EDT film with different air-exposure time.

Table S2 The parameters used for deconvolution of the S2p core level spectra of the PbS-EDT film.

component	peak (eV)	FWHM (eV)
PbS	160.7	1.2
	161.8	1.2
bound thiolate	161.6	1.4
	162.8	1.4
unbound thiol	163.6	1
	164.8	1

Extraction of diode ideality factors

According to the diode model, the dark *J-V* equation is:

$$J_d = J_0 \left[exp\left(\frac{qV}{nk_0T}\right) - 1 \right] \approx J_0\ exp\left(\frac{qV}{nk_0T}\right) \qquad (1)$$

Where q is the elementary charge, k_0T the thermal energy, J_d the dark current density, J_0 the saturation dark current intensity, n the ideality factor. By taking natural logarithm of the equation (1), equation (2) can be obtained, as follows,

$$ln(J_d) = ln(J_0) + \frac{qV}{nk_0T} \qquad (2)$$

Equation (2) displays the linear relationship between $In(J_d)$ and V, where q/nk_0T is the slope of the curve, q/k_0T is a constant (38.46 at room temperature). According to the slope of equation (2), the ideality factor (n) can be extracted directly.

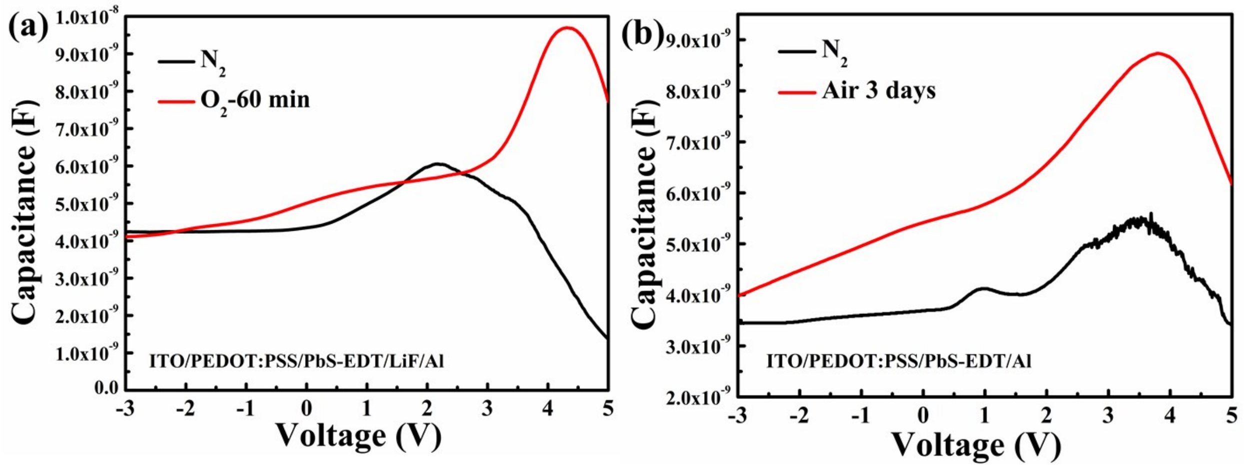

Fig.S5 (a) Capacitance-Voltage curves of the device with a structure of ITO/PEDOT:PSS/PbS-EDT/LiF/Al before air exposure and after one hour of oxygen exposure. (b) Capacitance-Voltage curves of the device with a structure of ITO/PEDOT:PSS/PbS-EDT/Al before and after air exposure.
